# A novel experimental approach to study disobedience to authority

**DOI:** 10.1038/s41598-021-02334-8

**Published:** 2021-11-25

**Authors:** Emilie A. Caspar

**Affiliations:** 1 Moral and Social Brain Lab, Department of Experimental Psychology, Ghent University, Henri Dunantlaan, 2, 9000 Ghent, Belgium; 2 Center for Research in Cognition and Neuroscience, Université Libre de Bruxelles, Brussels, Belgium

**Keywords:** Human behaviour, Decision

## Abstract

Fifty years after the experiments of Stanley Milgram, the main objective of the present paper is to offer a paradigm that complies with up-to-date ethical standards and that can be adapted to various scientific disciplines, ranging from sociology and (social) psychology to neuroscience. Inspired by subsequent versions of Milgram-like paradigms and by combining the strengths of each, this paper presents a novel experimental approach to the study of (dis)obedience to authority. Volunteers are recruited in pairs and take turns to be ‘agents’ or ‘victims’, making the procedure fully reciprocal. For each trial, the agents receive an order from the experimenter to send a real, mildly painful electric shock to the ‘victim’, thus placing participants in an ecological set-up and avoiding the use of cover stories. Depending on the experimental condition, ‘agents’ receive, or do not receive, a monetary gain and are given, or are not given, an aim to obey the experimenter’s orders. Disobedience here refers to the number of times ‘agents’ refused to deliver the real shock to the ‘victim’. As the paradigm is designed to fit with brain imaging methods, I hope to bring new insights and perspectives in this area of research.

## Introduction

The experiment of Stanley Milgram is one of the most (in)famous in psychology^1^, within and beyond academia. Several variables account for this notoriety, such as the method used, the ethical issues associated, the enthralling results or the societal impact of the research topic. Milgram’s classical studies famously suggested a widespread willingness to obey authority, to the point of inflicting irreversible harm to another person just met a few minutes before. Beyond the studies of Milgram, the history of nations is also plagued by horrendous acts of obedience that have caused wars and the loss of countless lives^2^. History has fortunately shown that some individuals do resist the social constraint of receiving orders when their own morality is of greater importance than the social costs associated with defying orders (e.g.,^3,4^). To understand the factors that prevent an individual from complying with immoral orders, research on disobedience should focus on two main axes: (1) what social and situational factors support disobedience and (2) what individual differences support disobedience.

The first axe has already been largely investigated in past studies. From Milgram’s studies, important situational factors supporting disobedience have already been established^5^. For instance, disobedience increases if the experimenter is not physically present in the room or if two experimenters provide opposing views regarding the morality of the experiment. Subsequent versions and interpretations of Milgram’s studies^6–8^ as well as historical research^4,9^ also suggested the importance of several social (e.g. presence of a supporting group) and situational factors (e.g. family history, proximity with the ‘victim’, intensity of the pain; money) supporting resistance to immoral orders. However, the second axe regarding individual differences has been less systematically approached. A few studies^10,11^ previously explored personality traits that may influence disobedience (e.g. empathic concern, risk-taking) but most of these studies, however, have used relatively weak and potentially biased methods, such as self-reported questionnaires and methods based on cover stories. These studies are not sufficient to explain why, in a given situation, some people will refuse immoral orders and rescue threatened human beings while others will comply with such orders. With the current literature on disobedience, we have no idea about which neuro-cognitive processes drive inter-individual differences regarding the degree of disobedience. This aim could be achieved by offering a novel experimental approach that would make it possible to use novel techniques that give us a more direct access to the functioning of the brain and cognition, such as functional near-infrared spectroscopy (fNIRS), electroencephalography or Magnetic Resonance Imagery (MRI). Regrettably, the original paradigm and those bearing close similarity are not adapted to reliably answer those questions as they were not designed to fit with neuroimaging measurements. By combining the strengths of previous work on disobedience into a single experimental paradigm and adapting it to fit with cognitive and brain imaging measurements, this novel experimental approach could help to better understand, together with individual, social, and cultural factors, which mechanisms make it possible for an individual to refuse to comply with immoral orders.

There were several challenges to consider in order to develop such a paradigm, both ethical and methodological. Studying obedience and resistance to immoral orders involves putting volunteers in a situation where they have to make a decision on whether or not to commit ‘immoral acts’ under orders. A balance has to be found between what is acceptable from an ethical perspective and what is necessary for the research question. Milgram’s studies on obedience raised undeniable ethical issues^12–14^, mostly associated with high stress and the use a cover story, which involves deception. Some variants of Milgram’s studies were realized with immersive virtual reality to prevent the ethical issues associated with Milgram’s paradigm^15^, but the transparency of the fake scenario presented to participants does not capture decision-making in an ecological set-up. Other Milgram-based variants, such as the 150-V method, appear to replicate Milgram’s results^16^ with respect to the actual ethical standards, but methodological concerns are still present^17^ as cover stories are still used, which lead to interpretation issues. Beyond ethical considerations, the use of deception also indeed involves a doubt about whether or not volunteers truly believed the cover story. As a consequence, a reasonable doubt remains on how to interpret the results and this is one of the main critics associated with Milgram’s studies and following versions. Recent work on the reports of Milgram’s volunteers suggested that there are no strong and reliable evidence that participants believed in the cover story^8,14,18^. Others suggested that since the stress of participants was visible on video recordings during the experiment (e.g. hand shaking, nervousness), this suggests that participants actually believed that they were torturing another human being^19^. However, this interpretation has been challenged by another study showing that participants can have physiological reactions to stress even in an obviously-fake experimental set-up^15^. These contrasting interpretations of Milgram’s studies actually reinforce the idea that results can hardly be interpreted when cover stories are used^20^. To answer those criticisms, a real scenario had thus to be created, where participants made decisions that have real consequences on another human being.

An additional challenge is that methods relying on the original paradigm of Milgram, such as the virtual reality version^15^ or the 150-V method^16^ are not adapted to neuroimaging measurements. More specifically, with such Milgram-like experimental approaches, only a single trial would be recorded for the entire experimental session, that is, when the volunteer stops the experiment (if this happens). For cognitive and neuroimaging data collection, a single trial per participant is not a reliable result, which requires the averaging of several trials to obtain a good signal-to-noise ratio.

Another challenge at the methodological and conceptual levels it that several experimenters^1,5,21,22^ including myself^21–27^, noted that volunteers are extremely obedient when coming to an experiment. Personally, I have tested about 800 volunteers to investigate the mechanisms by which coercive instructions influence individual cognition and moral behaviors. For instance, by using behavioral, electrophysiological and neuroimaging methods, we have observed that when people obey orders to send real shocks to someone else, their sense of agency^23^, their feeling of responsibility^28^, empathy for the pain of the victim and interpersonal guilt^26^ are attenuated compared to a situation where they are free to decide which action to execute. Out of 800 volunteers tested, only 27 disobeyed my orders (i.e. 3.3%): 21 for prosocial reasons (i.e. they refused to administer an electric shock to another individual), 3 by contradiction (i.e. by systematically pressing the other button, not matter the content of the order), and 3 for antisocial reasons (i.e. by administering shocks despite my order not to do so). Although convenient to study how obedience affects cognition, this rate is indubitably an issue when studying disobedience. If participants almost never disobey, we can’t study the mechanisms through which resistance to immoral orders may develop in a given situation. Several reasons for not disobeying the experimenter’s orders have been suggested. Some consider that being obedient is part of the human nature as massive and destructive obedience has been observed through countless historical events^2^. Another current view on the experiments of Milgram is that volunteers were actually happy to participate and to contribute to the acquisition of scientific data^17^, thus explaining the high obedience rate observed. This effect has been referred to as ‘engaged followership’^29^. If that interpretation is correct, the volunteer’s willingness to come and help the experimenter acquiring scientific data creates an extra difficulty to obtain disobedience in an experimental setup. However, this interpretation is challenged by several studies reported by Milgram, which displayed a higher disobedience rate than his original study. For instance disobedience increases when the shocks’ receiver sits in the same room as the participant or when the authoritative experimenter is not physically present in the room^5^. If participants were indeed only guided by their willingness to help to acquire scientific data, this should be the case in any experimental set-up. As some studies involve a higher disobedience rate compared to the initial version of Milgram’s study^1^, they could thus, at a first glance, be used for studying disobedience. However, even if some versions of the initial study of Milgram offer a highly disobedience rate, thus making it possible to study the mechanisms through which resistance to immoral orders may develop in a given situation, these experimental set-ups are still not adapted for cognitive and neuroimaging measurements and still rely on the use of a cover story.

Taking all the presented challenges into account (i.e. not using cover stories to avoid interpretation issues; obtaining a fair rate of disobedience; using an experimental approach that also fits with cognitive and neuroimaging measurements; respecting ethical standards), the present paper presents a set of experiments that combine the strengths of past experimental work on (dis)obedience. Volunteers were openly involved and active (= real social situation) rather than having to act in fictitious scenarios (= imagined social situation, e.g. Slater et al., 2006). They were confronted with moral decisions to follow or not the orders from an experimenter to inflict a real painful shock to a ‘victim’ in exchange (or not) for a small monetary gain, thus avoiding the use of cover stories. Since the aim here is to develop a paradigm that could be used both in behavioral and neuroimaging studies, some basic characteristics had to be considered. For instance, to fit with a Magnetic Resonance Imagery (MRI) scanning environment, neither the ‘victim’, nor the experimenter were in the same room as the agent. A real-time video was thus used to display a video of the victim’s hand receiving shocks on the agent’s screen and headphones were used so the participant could hear the experimenter’s orders.

Another method to study disobedience would be to select participants who are more likely to disobey than others. Each volunteer was thus also asked to complete a series of personality questionnaires to evaluate if a specific profile is associated with a greater prosocial disobedience rate. Systematic post-experimental interviews were conducted at the end of each experiment in order to understand the decisions of volunteers to follow or not the orders of the experimenter and to ask them how they felt during the experiment.

## Method

### Participants

A hundred eighty naive volunteers (94 females) were recruited in same gender dyads (= 90 dyads). During the recruitment procedure, I ensured that the participants in each dyad were neither close friends (by mixing people studying different academic courses), nor relatives. To estimate the sample size a priori, I calculated the total sample size based on an effect size f of (0.3). To achieve a power of 0.85 for this effect size, the estimated sample size was 168 for 6 groups^30^. I increased the sample size slightly to 180 in order to prevent loss of data in case of withdrawals. Volunteers were randomly assigned to one of the 6 variants of the task (N = 30/variant). One volunteer was not taken into account because they only played the role of the ‘victim’ to replace a participant who did not show up. No volunteers withdrew from the experiment. For the remaining 179 volunteers, the mean age was 22.63 years old (SD = 2.77, range:18–35). A Univariate ANOVA with Age as the dependent variable and Variant as the fixed factor confirmed that age of the volunteers did not differ between the different variant of the tasks (*p* > 0.1, BF_10_ = 0.167). Volunteers received between €10 and €19.60 for their participation. All volunteers provided written informed consent prior to the experiment. The study was approved by the Ethics Committee of the Erasme Hospital (reference number: P2019/484). All methods were performed in accordance with the relevant guidelines and regulations.

### Method and Material

Six experimental set-ups were created in a between-subject design. In all six set-ups, volunteers were invited by pairs. One person was assigned to start as agent and the other one to start as ‘victim’. Their roles were switched mid-way, ensuring reciprocity. Compared to the experimental design of Milgram, both volunteers were real participants, not confederates. The reciprocity also avoided volunteers to be stuck in the role of the person providing pain to the other, thus attenuating the potential psychological distress of being in a perpetrator role only. Volunteers were given the possibility to choose the role they wanted to start with. In the case none of them had a preference, role assignment was decided by a coin flip, but volunteers were reminded that they could still decide themselves. This procedure allows to ensure that participants do not think that this procedure is a trick.

**Figure 1:**
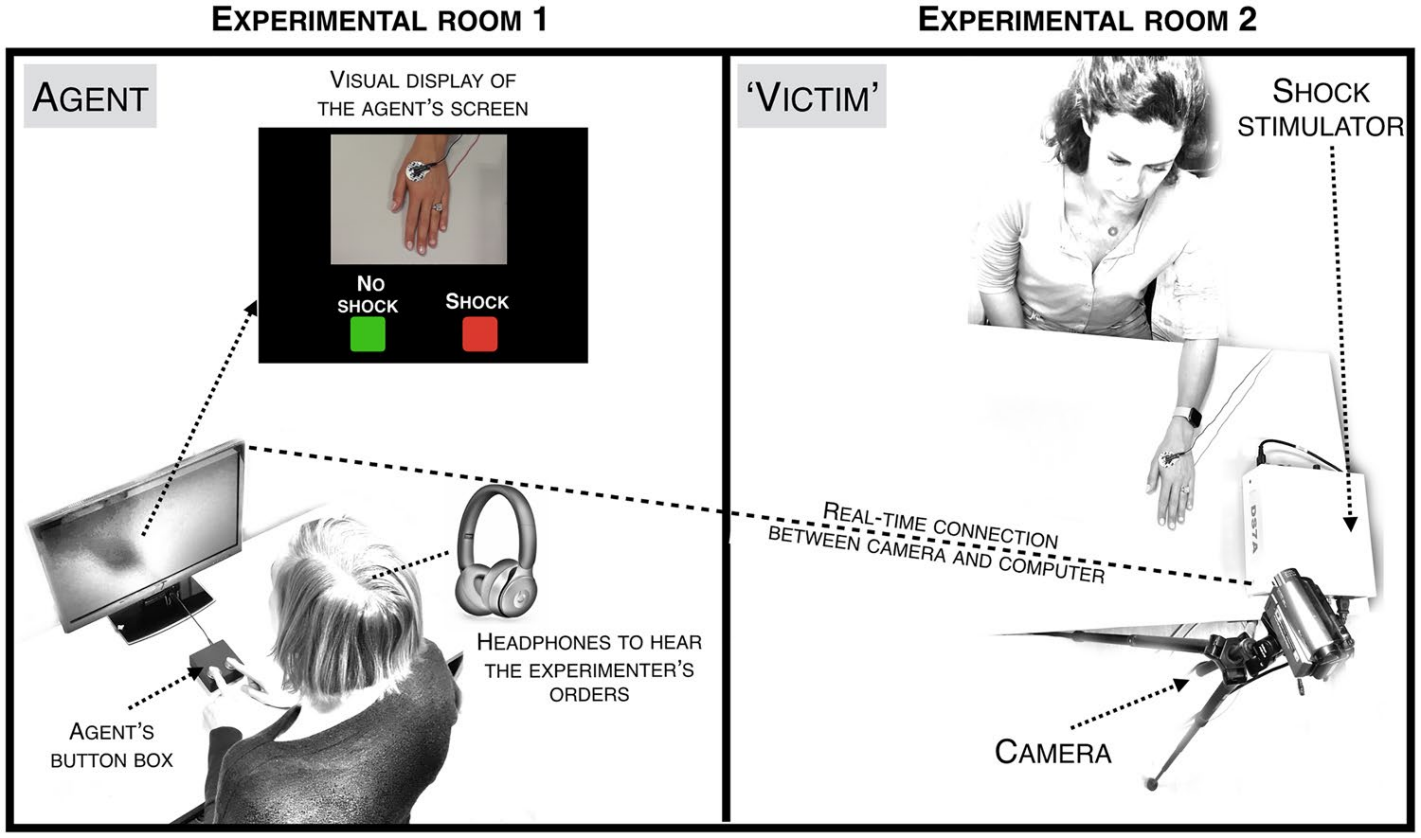
Experimental setup. Schematic representation of the experimental setup. Volunteers were in different rooms. The experimenter was located in a third, separated room. The agent heard on a trial basis the orders of the experiment through headphones and had to decide to press the ‘SHOCK’ or ‘NO SHOCK’ button. A real-time camera feedback displayed the hand of the victim of the agent’s screen so to allow to keep track on the consequences of their actions.

Volunteers were first given the instructions of the task. Then, they signed the consent forms in front of each other, so both were aware of the other’s consent. The experimenter was never present in the same room, but rather gave the instructions through headphones. This was for two reasons. First, Milgram’s studies show that disobedience increases if the experimenter is not physically present in the room. Second, in the case of MRI scanning, the experimenter would not be able to give direct verbal instructions to the volunteers in the MRI room due to the high noise of the scanner. Here, agents were isolated in a room and were provided headphones to hear the experimenter’s instructions (see Fig. 1). They were told that this was done to avoid attentional interferences through the experimenter’s physical presence in the room. In this series of studies, instructions were pre-recorded but a real setup with a microphone connected to the headphones could also work. Pre-recordings allow perfect timing of the events, important for neuroimaging or electroencephalography recordings. The instructions were “*give a shock*” or “*don’t give a shock*”. To increase the authenticity of the procedure, each sentence was recorded 6 times with small variations in the voice and displayed randomly. In addition, the audio recordings included a background sound similar to interphone communications.

Shocks were delivered using a constant current stimulator (Digitimer DS7A) connected to two electrodes placed on the back of victims’ left hand, visible to the agent through the camera display. Individual pain thresholds were determined for the two volunteers before starting the experiment. This threshold was determined by increasing stimulation in steps of 1 mA (Caspar et al., 2016). I approximated an appropriate threshold by asking a series of questions about their pain perception during the calibration (1. « *Is it uncomfortable?* »—2. « *Is it painful?* »—3. « *Could you cope with a maximum of 100 of these shocks?* »—4. « *Could I increase the threshold?* »). When roles were reversed, I briefly re-calibrated the pain threshold of the new victim by increasing the stimulation again from 0 in steps of 3 mA up to the previously determined threshold, to confirm that the initial estimate was still appropriate, and to allow re-familiarisation. The mean stimulation level selected by this procedure was 36.3 mA (SD = 17.5, V = 300, pulse duration: 200 µs). I chose this instead of other types of pain (e.g. financial) because it produces a clear muscle twitch on the victim’s hand each time a shock is sent. This allows volunteers to have a clear and visible feedback of the consequences of their actions and to be fully aware that shocks were real.

There was a total of 96 trials per experimental condition. In the coerced condition, the experimenter asked to give a shock in 64 trials and asked not to give a shock 32 trials. This ratio was chosen on the assumption that the volunteer’s willingness to refuse immoral orders would increase with the number of times they were instructed to inflict pain to the “victim”.

On each trial, a picture of two rectangles, a red one labelled ‘SHOCK’ and a green one labelled ‘NO SHOCK’, was displayed in the bottom left and right of the screen. The key-outcome mapping varied randomly on a trial-wise basis, but the outcome was always fully congruent with the mapping seen by the participant. Agents could then press one of the two buttons. Pressing the SHOCK key delivered a shock to the victim while pressing the NO SHOCK key did not deliver any shocks. This procedure of randomized button mapping allows to have a better control over motor preparation, an aspect that can be important for neuroimaging data.

In half of the variants of the task (i.e., 3/6), the “Aim” variants, participants were given a reason for obeying the orders of the experimenter, while this was not the case in the other half, the “No aim” variants. In the “No Aim” variants, I did not provide any reasons for obeying to the participants and I simply explained the task. If participants asked about the aim, I simply told them that they would know at the end of the experiment, without providing further justifications. In the “Aim” variants, volunteers were told that researchers observed a specific brain activity in the motor cortex in another study when participants were given instructions. We explained that the present study was a control study to measure different aspects linked to motor activity when they press buttons, in order to see if the button pressing was related to brain activity measured over the motor cortex. To increase the veracity of the aim, electrodes were also placed on their fingers and connected to a real electromyography (EMG) apparatus to supposedly record their muscle activity. Volunteers were instructed to press the two buttons only with their right and left index fingers, as naturally as possible, and to avoid producing too ample movements to create clean EMG data. In the case volunteers asked if they really had to follow orders, I told them that for ethical reasons I could not force them to do anything, but that it would be better for the sake of the experiment. Telling them explicitly that they could disobey the orders would not be beneficial in the quest of studying ‘real’ disobedience.

In 4 out of 6 variants of the task, the “Free-choice” variants, a second experimental condition was used, the free-choice condition. In this condition, volunteers were told that they could freely decide in each trial to shock the ‘victim’ or not. In this condition, they did not receive instructions. In 4 out of 6 variants of the task, the “Monetary reward” variants, agents received a monetary reward of + €0.05 for each shock delivered. In the other 2 variants, volunteers were not rewarded for each shock delivered (i.e. “No monetary reward” variants). To resume, the 6 variants of the same task were the following: (1) No Aim + Monetary reward + Free-choice condition; (2) No Aim + No monetary reward + Free-choice condition; (3) Aim + Monetary reward + Free-choice condition; (4) Aim + No monetary reward + Free-choice condition; (5) No Aim + Monetary reward + No free-choice condition; (6) Aim + Monetary reward + No free-choice condition (see Table 1).

**Table 1:** Schematic representation of each variant of the experimental task.

Variants of the task	Aim for obedience	Monetary reward	Free-choice condition
Variant 1	✗	✓	✓
Variant 2	✗	✗	✓
Variant 3	✓	✓	✓
Variant 4	✓	✗	✓
Variant 5	✗	✓	✗
Variant 6	✓	✓	✗

Before the experimental session, volunteers filled in six questionnaires. Those questionnaires included (1) the Money Attitude Scale (e.g. “*I put money aside on a regular basis for the future*”)^31^, (2) the Moral Foundation Questionnaire (e.g. “*Whether or not someone showed a lack of respect for authority*”)^32^, (3) the Aggression-Submission-Conventionalism scale (e.g., “*We should believe what our leaders tell us*”)^33^, (4) the short dark triad scale (e.g., “*Most people can be manipulated*”)^34^, the Interpersonal Reactivity Index (e.g. “*When I see someone get hurt, I tend to remain calm*”)^35^. At the end of the experimental session, they were asked to fill in two more questionnaires: (1) A debriefing assessing what they felt during the experiment and the reasons for choosing to obey or disobey the orders of the experimenter (Supplementary Information S1) and (2) a questionnaire on social identification with the experimenter (e.g., “*I feel strong ties with this experimenter*”)^36^. At the end of the experiment a debriefing was conducted for each volunteer, separately. Volunteers were then paid, again separately.

### General data analyses

Each result was analyzed with both frequentist and Bayesian statistics^37^. Bayesian statistics assess the likelihood of the data under both the null and the alternative hypothesis. BF_10_ corresponds to the *p*(data|*H*_1_)/*p*(data|*H*_0_). Generally, a BF between 1/3 and 3 indicates that the data is similarly likely under the H_1_ and H_0_, and that the data does not adjudicate which is more likely. A BF_10_ below 1/3 or above 3 is interpreted as supporting H_0_ and H_1_, respectively. For instance, BF_10_ = 20 would mean that the data are 20 times more likely under H_1_ than H_0_ providing very strong support for H_1_, while BF_10_ = 0.05 would mean that the data are 20 times more likely under H_0_ than H_1_ providing very strong support for H_0_^38^. BF and p values were calculated using JASP^39^ and the default priors implemented in JASP. All analyses were two-tailed.

## Results

### Number of shocks given in the free-choice condition

In the free-choice condition, volunteers were told that they were entirely free to decide to deliver a shock or not to the ‘victim’ on each of the 96 free-choice trials. On average, agents administered shocks to the victim on 31.86% of the trials (SD = 34.98, minimum: 0%, maximum: 100%) in the free-choice condition, corresponding to 30.59/96 shocks. A paired-sample t-test indicated that agents delivered less frequently a shock in the free-choice condition than in the coerced condition (68.03%, SD = 41.11, t_(119)_ = -9.919, *p* < 0.001, Cohen’s d = − 0.906, BF_10_ = 1.987e + 14). This result supports the fact that individuals can inflict more harm to others when they obey orders than when they act freely.

### Prosocial disobedience across variants

In the present study, I was interested in prosocial disobedience, that is, when agents refuse the orders of the experimenter to send a painful shock to the ‘victim’. Table 2 displays the number of volunteers who reported that they voluntarily disobeyed in each variant of the task.

**Table 2:** Number of volunteers who reported that they voluntarily disobeyed the orders of the experimenter.

	Variant 1	Variant 2	Variant 3	Variant 4	Variant 5	Variant 6
Voluntary disobedience (‘Yes’)	23/30	24/30	8/30	16/30	24/30	13/30

**Figure 2:**
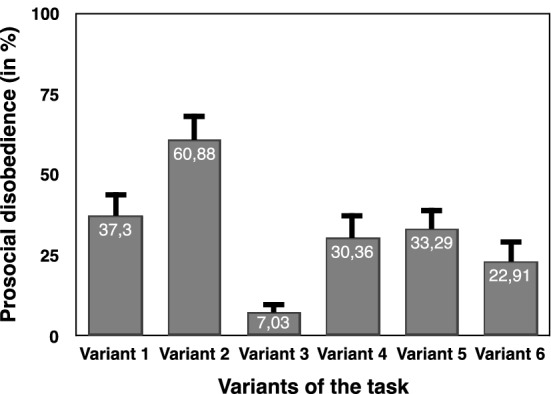
Graphical representation of the percentages of prosocial disobedience in each variant of the task.

In this experiment, the main variable of interest was not to consider how many participants disobeyed in each variant only, but also how *frequently* they disobeyed. A percentage of prosocial disobedience was calculated for each volunteer, corresponding to the number of trials in which participants chose to disobey (i.e., sending no shocks while ordered by the experimenter to do so) divided by the total number of trials corresponding to the order to send a shock, multiplied by 100. I compared the prosocial disobedience rate across variants of the task, gender of participants and order of the role. I conducted a univariate ANOVA with prosocial disobedience as the dependent variable and Aim (aim given, no aim given), Monetary reward (+ €0.05 or not), Free-choice (presence or absence of a free-choice condition), Gender and Order of the Role (agent first, victim first) as fixed factors (see Fig. 2). Both frequentist and Bayesian statistics strongly supported a main effect of Aim (F_(1,155)_ = 14.248, *p* < 0.001, η^2^_***partial***_ = 0.084, BF_incl_ = 158.806). Prosocial disobedience was lower when an aim for obedience was given to volunteers (20.4%, CI_95_ = 12.8–28.1) than when no aim was given (43.3%, CI_95_ = 35.6–51). Both frequentist and Bayesian statistics also supported a main effect of Monetary reward (F_(1,155)_ = 12.335, *p* = 0.001, η^2^_***partial***_ = 0.074, BF_incl_ = 28.930). Prosocial disobedience was lower when a monetary reward was given for each shock (25.1%, CI_95_ = 18.5–31.7) than when no monetary reward was given (45.4%, CI_95_ = 35.9–54.8). The frequentist approach showed a main effect of Gender (F_(1,155)_ = 5.128, *p* = 0.025, η^2^_***partial***_ = 0.032), with a lower prosocial disobedience rate for female volunteers (25.7%, CI_95_ = 18.2–33.2) then for male volunteers (38%, CI_95_ = 30–46). However, the Bayesian version of the same analysis revealed a lack of sensitivity (BF_incl_ = 0.871). All other main effects or interactions supported H_0_ or a lack of sensitivity (all *p*s > 0.1 & BFs_incl_ ≥ 0.4.291E-7 & ≤ 1.178).

The following results report two-tailed Pearson correlations between prosocial disobedience and several other variables, including (1) the reasons given for disobeying, (2) the feeling of responsibility, badness and how sorry they experienced during the experiment, (3) the identification with the experimenter, (4) the perceived level of pain of the victim, (5) identification with the ‘victim’, and (6) individual differences measured through self-report questionnaires. I applied a False Discovery Rate (FDR) approach with the Benjamini and Hochberg method^40^ to each p-value for each of those correlations but for the sake of clarity these variables are reported in different sub-sections.

### Reasons for prosocial disobedience

All participants who reported that they voluntarily disobeyed the orders of the experimenter (N = 108) were presented a list of 10 reasons that they had to rate from “Not at all” to “Extremely” (see Supplementary Information S1). The reason ‘*I wanted to make more money*’ was only considered for the data of volunteers who had a variant with a monetary reward for each shock (N = 68). Both frequentist and Bayesian statistics showed that the percentage of prosocial disobedience positively correlated with moral reasons (r = 0.550, *p*_*FDR*_ < 0.001, BF_10_ = 1.700e + 7), positively correlated with disobedience by contradiction (r = 0.329, *p*_*FDR*_ < 0.001, BF_10_ = 47.53) and negatively correlated with the willingness to make more money (r = − 0.485, *p*_*FDR*_ < 0.001, BF_10_ = 822.16). Other correlations were in favor of H_0_ or were inconclusive (all *p*s_*FDR*_ > 0.076, all BFs_10_ ≥ 0.120 & ≤ 1.446).

### Feeling responsible, bad and sorry

**Figure 3:**
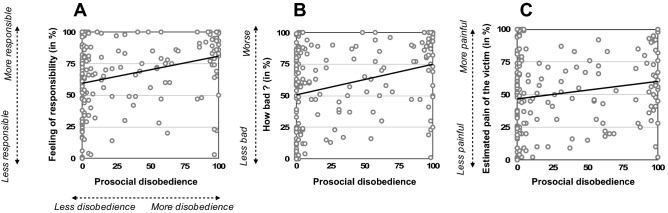
Graphical representation of Pearson correlations between prosocial disobedience and (**A**) feeling of responsibility, (**B**) how bad agents felt during the task when they administered shocks to the ‘victim’, and (**C**) how painful they estimated the shock delivered to the ‘victim’ was. All tests were two-tailed.

Both frequentist and Bayesian statistics showed strong positive correlations between prosocial disobedience and how responsible (r = 0.299, *p*_*FDR*_ < 0.001, BF_10_ = 343.98) and how bad (r = 0.301, *p*_*FDR*_ < 0.001, BF_10_ = 384.65) they felt during the task (see Figs. 3A and B). The more responsible and worse they felt during the task, the more they refused the order to send a shock to the ‘victim’. How sorry they felt was inconclusive (*p*_*FDR*_ > 0.08, BF_10_ = 0.929).

### Identification with the experimenter

Both frequentist and Bayesian statistics strongly supported H_0_ regarding the relationship between prosocial disobedience and personal identification (*p*_*FDR*_ > 0.5, BF_10_ = 0.121) and bonding with the experimenter (*p*_*FDR*_ > 0.5, BF_10_ = 0.117). The relationship between the charisma of the experimenter and prosocial disobedience was also slightly in favor of H_0_ (*p*_*FDR*_ > 0.1, BF_10_ = 0.530).

### Estimated pain of the ‘victim’

The frequentist approach showed a positive correlation between the perceived pain of the ‘victim’ and prosocial disobedience (r = 0.189, *p*_*FDR*_ = 0.048). The higher they considered the ‘victim’ to be in pain, the more frequently they refused to deliver the shock. The Bayesian version of the same analysis slightly supported this relationship (BF_10_ = 2.236), see Fig. 3C.

### Identification with the ‘victim’

In the post-session questionnaire, volunteers had to identify to what extent they considered that the other participant could be part of their group and to what extent they identified with the other participant. Both frequentist and Bayesian statistics strongly supported H_0_ regarding the relationship between prosocial disobedience and the perception that the other participant could be part of one’s own group (*p*_*FDR*_ > 0.8, BF_10_ = 0.096). The relationship between prosocial disobedience and the identification with the other participant also slightly supported H_0_ (*p*_*FDR*_ > 0.1, BF_10_ = 0.511).

### Correlations between the behavior of pairs of participants

As we used a role reversal procedure, the behavior of those who were agents first could influence the behavior of those who turned agents afterwards. A Pearson correlation between prosocial disobedience of agents first and prosocial disobedience of victims who turned agents afterwards. The correlation was positive (r = 0.514, *p* < 0.001, BF_10_ = 60,068.704), suggesting participants who were agents second tend to act similarly as those who were agents first.

### Individual differences associated with prosocial disobedience

Another approach to ensure a reliable prosocial disobedience rate when recruiting volunteers would be to target individuals with a profile that is most frequently associated with disobedient behaviors. Both frequentist and Bayesian statistics for exploratory correlations were two-tailed. Cronbach’s α for each subscale is presented in Supplementary Information S2. Both frequentist and Bayesian statistics showed a negative correlation between scores on the Authority subscale (r = -0.259, *p*_*FDR*_ < 0.001, BF_10_ = 41.372) and the Purity subscale (r = -0.303, *p*_*FDR*_ < 0.001, BF_10_ = 424.97) from the MFQ questionnaire. The lower volunteers scored on authority and purity, the higher was their prosocial disobedience rate. Other correlations were in favor of H_0_ or were inconclusive (all *p*s_*FDR*_ ≥ 0.048, all BFs_10_ ≥ 0.100 & ≤ 2.314).

### Reasons for obedience

If participants reported that they did not voluntarily disobey the orders of the experimenter, they were asked in an open question to explain their decision to comply with those orders. After reading all the answers, three categories were extracted from the reasons provided: (1) ‘For science’ reasons; participants reported that they obeyed to allow reliable data acquisition (e.g., Participant 91: “*Pour ne pas fausser l’étude*”—English translation: “*To avoid biasing the stud*y”); (2) ‘For respect of authority’ reasons; participants reported that they had to follow the orders of the authoritative figure (e.g., Participant 13: “*Pour moi c’est normal de suivre un ordre*”—English translation: “*In my opinion, it’s normal to follow an order*”), and (3) ‘For lack of side-effects’ reasons; participants reported that since the shocks delivered were calibrated on one’s own pain threshold, obeying orders to shock was not problematic (e.g., Participant 115: “*Douleur supportable pour l'autre, je n'ai accepté de faire subir que ce que j'aurais été prêt à subir moi-même*”—English translation: “*The pain was tolerable for the other participant, I have accepted to inflict the intensity of the pain that I would have been ready to undergo myself*”). An independent, naive judge classified the response of participants in one or several of those three established categories. Analyses of the frequencies revealed that the reason “For Science” was mentioned 31/70 times, the reason “For lack of side-effects” was mentioned 17/70 times and the reason “For respect of authority” was mentioned 31/70 times.

## Discussion

The aim of the present paper was to present a novel experimental approach to study (dis)obedience to immoral orders, by combining the strength of past experimental work and by adapting it to cognitive and neuroimaging measurements. Although other versions were proposed since Milgram’s studies, like a study in an immersive virtual environment^15^ or the 150-V method^16^, some methodological concerns remained as those methods still involved cover stories or fake experimental set-ups. Here, the experimental approach was significantly different as it was based on an entirely transparent method that involved the administration of real electric shocks to another individual. This approach has the advantage to solve some of the main ethical and methodological concerns associated with the use of cover stories. It also has the advantage that it be can used both to study how social and situational factors influence disobedience as well as individual factors. For social and situational factors, the proposed paradigm can be adapted to evaluate for instance the influence of a supporting group, the use of high or low monetary rewards or how priming disobedience with a documentary influence disobedience. For individual factors, the paradigm allows to investigate how personality traits influence disobedience or to study the neuro-cognitive processes underlying disobedience.

Some novel theories combining a multi-method approach based on social psychology, neuroeconomics and neuroscience could thus emerge to understand better the mechanisms supporting disobedience. For instance, one could evaluate how empathy for the pain of the victim predicts disobedience and how the presence of a supporting group influences our capacity to feel empathy^41^ and/or compassion for the ‘victim’^42^. It could also be argued that the presence of a supporting group diffuses responsibility between individuals and increases obedience, by influencing how our brain processes agency and responsibility over our actions^28,43–45^. As the results obtained in the present study also indicated that feeling bad for the shocks delivered was statistically associated with prosocial disobedience, one could evaluate how the neural correlates of guilt^46^ predicts prosocial disobedience and what historical, cultural and individual factors influence the feeling of guilt.

Six variants of the same task were tested in the present study, some inducing a higher prosocial disobedience rate than others. Statistical results showed that providing a reason—or aim—to justify obedience strongly decreased disobedience. Providing a monetary reward, even one as small as €0.05, also strongly decreased disobedience. Variant 2, in which volunteers were not given an aim or monetary reward, showed the highest disobedience rates. However, to study disobedience in ecological way, the paradigm should capture disobedience of participants even if they know that they are losing something (i.e., monetary rewards or the ‘trust’ of the experimenter asking them help for the study). Defying the orders of an authority generally involves social and/or monetary costs in real-life situations. I would thus not recommend using an experimental paradigm in which volunteers have no costs associated with defying the orders of the experimenter, as it would reduce the ecology of the disobedience act. Variants 3 and 6 involve two types of costs for resisting the orders of the experimenter: a monetary loss and deceiving the experimenter. In Variant 3, descriptive statistics showed that prosocial disobedience was lower compared to Variant 6. The main difference between these two variants was the presence of a free-choice condition. In my former studies^23,27^, volunteers frequently justified obedience in the coerced condition because they were given freedom in the free-choice condition (e.g. Participant 89 – English Translation: “ *(…) In addition, I knew I could chose freely in the other condition not to send shocks—what I did*). In the present debriefings, some volunteers also reported that the presence of a free-choice condition was giving them enough freedom to accept to follow the orders in the coerced condition. In the supplementary analyses, results showed that when the monetary reward and the aim for obeying are identical, being given a free-choice condition reduces disobedience in the coerced condition. Therefore, Variant 6 appears to provide a good balance between reaching a reliable disobedience rate and finding volunteers who would refuse to produce physical harm on another human beings despite the monetary or social costs associated with defying orders.

Another approach would be to pre-select people who are predicted to be more disobedient. Personality questionnaires indicated that scoring low on the authority and on the purity subscale of the MFQ was strongly associated with a higher prosocial disobedience rate. The link between one’s own relationship to authority and prosocial disobedience observed here replicates another study conducted on the first generation of Rwandese after the 1994 genocide^47^. One’s own relationship to authority thus appears to be a reliable predictor variable in order to pre-select a sample that is more likely to disobey immoral orders.

In the present paper, administering a real mildly painful shock in exchange or not for a small monetary gain was described as an ‘immoral’ act. The notion of what is moral or not can highly differ between individuals^48^, for both academics and volunteers participating in an experiment. Humans are indeed sensitive to different competing issues of morality, a key reason for rescuing persecuted people^49^. In accordance with this observation, the present results indicated that moral reasons were a critical factor associated with the prosocial disobedience rate: the more shocking partners was considered as immoral, the more volunteers disobeyed. However, considering an action as against one’s own moral values does not necessarily translate to a refusal—especially when this order is in line with the Law. An extreme example is soldiers who have perpetrated acts that transgressed their moral beliefs but were issued by their superior in combat^50^. A core question for future research remains: Why are some people capable of putting their own moral standards above the social costs associated with defying orders?

Results indicate that the more volunteers felt responsible during the task, and the worse they felt for sending shocks to the ‘victim’, the higher was their prosocial disobedience. In another study, we observed that obeying orders reduced the feeling of responsibility, how bad and how sorry volunteers felt compared to being free to decide ^26^. One hypothesis is that individuals who have preserved a feeling of responsibility and feeling bad—even under command—could more easily defy immoral orders. However, future studies are necessary to better understand the neuro-cognitive processes that prevent an individual from complying with immoral orders. As this paradigm is adapted to neuroimaging measurements, a whole range of studies could now be conducted.

It has been previously suggested that a strong identification with the experimenter giving orders is associated with higher obedience^36^. However, in the present paper, correlations between prosocial disobedience and identification with the experimenter were in favor of H0 with both the frequentist and the Bayesian approaches. In a former study, we also observed that identification to the experimenter was not a critical aspect for explain (dis)obedience. We observed that the generation of Rwandese born after the genocide and tested in Rwanda reported a higher identification to the experimenter than the same generation of Rwandese but tested in Belgium^47^. However, the latter group had a higher prosocial disobedience rate than the former group. Future studies must thus be conducted to understand how the identification with the person giving orders could influence obedience and its weight compared to other social, cultural and individual variables.

Although some volunteers reported that they felt a bit stressed and anxious during the task when they were in the role of the agent, the overwhelming majority did not report any negative psychological feelings. None of the participants withdrew from the experiment and none reported long-term negative psychological effects.

Nowadays, it has become difficult to find volunteers who do not know Milgram’s studies given the high media coverage, including movies, radio soaps, books, podcast and documentaries. One could expect that knowing Milgram would prevent people to obey. However, for the large majority of volunteers, it appears that this is not the case. In previous studies that I conducted with a relatively similar paradigm, the disobedience rate was drastically low (i.e. 3.3%) even if participants were university students knowing Milgram’s studies. In the present study, almost all the volunteers who participated in the present study knew Milgram and explicitly mentioned him during the oral debriefings or before starting the experiment. Yet for those who disobeyed, almost none reported that the reason for disobedience was that they thought it was the aim of the experiment. Further, there was no statistical relationship between prosocial disobedience and believing that it was the aim of the study. It does not mean that knowing Milgram would not influence at all disobedience. It rather suggests that knowing Milgram is not the main factor influencing one’s decision to obey or not an experimenter. It is also possible that since in this experiment shocks were real and not fake such as in Milgram’s studies, participants considered that this was indeed not a study aiming to replicate Milgram.

As far as I have observed, the main problem associated with knowing Milgram’s studies is that volunteers believe that I also have hidden aims and procedures when they enter the experimental room. Several volunteers reported that they only realized that my explanations for the task were true when they were explicitly offered the choice to decide which role to play first and/or when they started receiving the shocks. This is a general concern in psychological studies: The high use of cover stories can also impact other research, as volunteers start to develop a mistrust in what researchers tell them.

Results indicated that who were agents second tend to act similarly as those who were agents first, by sending a relatively similar amount of shocks. Of note, this is an effect that we also observed in past studies on the effect of obeying orders on cognition^23,26,43^. Nonetheless, in none of those studies we observed that the order of the role had a statistical influence on the neuro-cognitive processes targeted. However, the influence on role reversal on disobedience and related neuro-cognitive processes has still to be investigated in future studies.

The present paradigm is ecological in the sense that volunteers are facing decisions that have a real, physical impact on another human being. However, at the moment I only have little evidences that this paradigm has ecological validity to reflect obedience in real life situations, especially regarding “destructive disobedience”^17^. Caution is indicated when making inference from laboratory studies to complex social behaviours, such as those observed during genocides^16^. My main evidence at the moment is that the very low rate of prosocial disobedience observed in the first generation of post-genocide Rwandans tested in Rwanda using this paradigm^47^ is consistent with the fact that deference to authority had already been emphasized by academics as an important factor in the 1994 genocide^4,51^. Individual scores on deference to authority in Caspar et al.^47^ was the best predictive factor for prosocial disobedience in that former paradigm, thus suggesting some ecological validity. A promising approach would be to recruit “Righteous Among the Nations”, individuals who really saved lives during genocides. Testing this population with the present paradigm would put the ecological validity of this paradigm to the test.

People’s ability to question and resist immoral orders is a fundamental aspect of individual autonomy and of successful societies. As Howard Zinn famously wrote: “*Historically, the most terrible things—war, genocide, and slavery—have resulted not from disobedience, but from obedience*”. Understanding how individuals differ in the extent to which they comply with orders has undeniably several societal implications. They range from understanding how evolving in highly hierarchical environments*—*such as the military or prisons—influences moral behaviours, to developing interventions that would help to prevent blind obedience and help to resist calls to violence in vulnerable societies. However, since Milgram’s studies, the topic of disobedience has been mostly studied by social psychologists using adapted versions of the initial paradigm developed by Milgram. I hope that with this novel approach, (dis)obedience research will be given a new boost and will be considered by other scientific disciplines seeking to understand better human behaviours.

## Supplementary Information

Supplementary Information.

## Data Availability

Data are made available on OSF (DOI: https://doi.org/10.17605/OSF.IO/2BKJC).
