# Supplementary material for: A novel experimental approach to study disobedience to authority
Source: Sci Rep. 2021 Nov 25;11:22927. doi: 10.1038/s41598-021-02334-8 (PMC8617051; doi:10.1038/s41598-021-02334-8)
Supplement: Supplementary file 1 — Supplementary Information. [file 41598_2021_2334_MOESM1_ESM.docx]

SUPPLEMENTARY INFORMATION S1. Post-session questionnaire

**Mettez un trait pour indiquer votre réponse. Cela se réfère à lorsque vous étiez AGENT.**

1. A quel point vous sentiez-vous responsable durant l’expérience ?

Pas du tout Extrêment

2. A quel point vous sentiez-vous mal d’envoyer un choc en échange d’argent ?

Pas du tout Extrêment

3. A quel point vous sentiez-vous désolé d’envoyer un choc à la victime ?

Pas du tout Extrêment

4. Indiquez à quel point vous pensiez que le choc était douloureux pour la victime ?

Pas du tout Extrêment

5. Avez-vous VOLONTAIREMENT désobéi aux ordres de l’expérimentateur ?

**OUI NON**

- Si vous avez indiqué **NON** à la question précédente, pouvez-vous expliquer les raisons qui vous ont poussé à suivre les ordres ?

__________________________________________________________________________________________________________________________________________________________________________________________________________________________________________________________________________________________________________________________________________________________________________________________________________________________________________________________________

- Si vous avez indiqué **OUI** à la question précédente, répondez aux questions suivantes sur les raisons de la désobéissance.

A. Je me sentais mal pour la victime

Pas du tout Extrêment

B. Il y avait trop de chocs

Pas du tout Extrêment

C. Je n’aime pas qu’on me dise quoi faire

Pas du tout Extrêment

D. Je voulais me faire plus d’argent

Pas du tout Extrêment

E. Je m’ennuyais et c’était un moyen de rendre la tâche plus intéressante

Pas du tout Extrêment

F. Je pensais que c’était le but de l’expérience

Pas du tout Extrêment

G. J’avais peur de me faire juger par les autres personnes présentes dans la salle

Pas du tout Extrêment

H. Mon éducation (familiale) a influencé mes décisions

Pas du tout Extrêment

I. L’histoire de mon pays d’origine a influencé mes décisions

Pas du tout Extrêment

J. Pour des raisons morales

Pas du tout Extrêment

K. Seulement pour les agents en premier. J’avais peur de recevoir plusieurs chocs en retour quand je passerai au rôle de victime

Pas du tout Extrêment

L. Seulement pour les victimes en premier. J’avais reçu de nombreux chocs en étant victime et cela m’a semblé juste

Pas du tout Extrêment

6. Si vous avez désobéi pour d’autres raisons, veuillez les indiquer ici :

_______________________________________________________________________________________________________________________________________________________________________________________________________________________________________________________________________________________________________________________________________________________________________________________

7. A quel point vous identifiez-vous à l’autre participant ?

0 1 2 3 4 5

(pas du tout) (totalement)

8. Considérez-vous que l’autre participant faisait partie de votre propre groupe ?

0 1 2 3 4 5

(pas du tout) (totalement)

9. S’il-vous-plait décrivez en quelques mots comment vous vous sentiez durant l’expérience

____________________________________________________________________________________________________________________________________________________________________________________________________________________________________________________________________________________________________________

SUPPLEMENTARY INFORMATION S2 – Cronbach’s α

| Questionnaire | Subscale | Cronbach’s α |
| --- | --- | --- |
| Identification to the experimenter | Personal Identification | .830 |
|  | Personal bond | .905 |
|  | Charisma | .916 |
| Interpersonal Reactivity Index | Empathic concern | .538 |
|  | Perspective-taking | .641 |
|  | Personal distress | .795 |
|  | Fantasy seeking | .725 |
| Short Dark Triad | Machiavelism | .723 |
|  | Narcissism | .589 |
|  | Psychopathy | .674 |
| Aggression-Submission-Conventionalism scale | Submission | .480 |
|  | Conventionalism | .650 |
|  | Aggression | .598 |
| Money Attitude scale | Power/Prestige | .856 |
|  | Retention/Time | .844 |
|  | Distrust | .834 |
|  | Anxiety | .656 |
| Moral Foundation Questionnaire | Harm | .670 |
|  | Fairness | .549 |
|  | Loyalty | .534 |
|  | Authority | .747 |
|  | Purity | .760 |
